# Supplementary material for: Psychological Correlates of Attitudes toward Pet Relinquishment and of Actual Pet Relinquishment: The Role of Pragmatism and Obligation
Source: Animals (Basel). 2019 Dec 29;10(1):63. doi: 10.3390/ani10010063 (PMC7022558; doi:10.3390/ani10010063)
Supplement: Supplementary file 1 [file animals-10-00063-s001.pdf]

Supplementary Material

PORTUGUESE QUESTIONNAIRE

Q97 Qual a sua idade?

____

Q98 Sexo:

- ○ Masculino (1)
- ○ Feminino (2)
- ○ Outro (3)

Q99 Qual o último grau de escolaridade que concluiu?

- ○ Inferior ao Ensino Secundário (liceu) (1)
- ○ Ensino Secundário (liceu) (2)
- ○ Bacherlato/Licenciatura (3)
- ○ Mestrado/Doutoramento (4)
- ○ Outro (5)

Q104 Qual a sua orientação política?

- ○ Extrema direita (1)
- ○ Direita (2)
- ○ Centro (3)
- ○ Esquerda (4)
- ○ Extrema esquerda (5)
- ○ Sem orientação política (6)

Q105 Qual a sua religião?

- ○ Sem religião (1)
- ○ Católica (2)
- ○ Cristã não católica (3)
- ○ Outras religiões (4)

Q100 Qual o seu nível de rendimento mensal:

- ○ < = 580€ (1)
- ○ 581-999€ (2)
- ○ 1000-1999€ (3)
- ○ 2000-4999€ (4)
- ○ = > 5000€ (5)

Q14 A sua habitação é partilhada ou vive sozinho/a?

- ○ Vivo sozinho/a (1)
- ○ Vivo com os meus pais (2)
- ○ Vivo com o meu/minha companheiro/a (3)
- ○ Vivo com amigos/as (4)
- ○ Outra situação: (5) ____

Supplementary Material

Q5 Qual o número de crianças que tem a seu cargo ou que habitam consigo?

- 0 (1)
- 1 (2)
- 2 (3)
- 3 (4)
- 4 (5)
- < 5 (6)

Q32 Faz voluntariado?

- Sim (1)
- Não (2)

Q101 Trabalha com animais?

- Sim (1)
- Não (2)

Q18 Já alguma vez teve animais de companhia?

- Sim (1)
- Não (2)

Q28 Quem decidiu ter animais?

- Fui eu (1)
- Foi um familiar (2)

Q26 Os seus amigos/familiares têm animais de companhia?

- Não, quase ninguém (1)
- Sim, alguns (2)
- Sim, quase todos (3)

Q20 Tem algum animal neste momento?

- Sim (1)
- Não (2)

Q36 É o cuidador principal deste animal?

- Sim, sou eu sozinho/a (1)
- Sim, mas tenho ajuda (2)
- Não (3)

Supplementary Material

Q70 O seu animal de companhia é, para si, um peso ou um fardo?

- ○ Nunca 1 (1)
- ○ 2 (2)
- ○ 3 (3)
- ○ 4 (4)
- ○ 5 (5)
- ○ 6 (6)
- ○ Sempre 7 (7)

Q72 Quais os motivos que o/a levariam a abdicar do seu animal?

	Discordo totalmente 1 (1)	2 3 4 5 6	Concordo totalmente 7 (7)
Não ter solução para as férias (1)			
Doença dispendiosa do animal (2)			
Idade avançada (3)			
Comportamentos agressivos (4)			
Comportamentos destrutivos (5)			
Urina ou fezes fora do sítio (6)			
Eu ou familiar alérgico ao animal (7)			
Personalidade do animal incompatível comigo (8)			
Personalidade do animal incompatível com a família (9)			
Comportamento do animal incompatível comigo (10)			
Comportamento do animal incompatível com a família (11)			

Q75 Já alguma vez teve de abdicar de um animal porque não o podia manter? O que aconteceu?

- ○ Nunca aconteceu (1)
- ○ Sim, entreguei o animal a um familiar/amigo (2)
- ○ Sim, devolvi o animal à origem (criador/associação/particular) (3)
- ○ Sim, vendi o animal (4)
- ○ Sim, soltei o animal no exterior (5)
- ○ Sim, o animal foi eutanasiado (6)
- ○ Sim, entreguei a uma associação de proteção animal (7)
- ○ Outro: (8) ____

Q77 Alguma vez se arrependeu de ter o seu animal de companhia?

- ○ Sim (1)
- ○ Não (2)

Supplementary Material

Start of Block: abandono

Q85 Leia estas afirmações e indique o quanto concorda ou discorda de cada uma:

	Discordo totalmente 1 (1)	2 3 4 5 6	Concordo totalmente 7 (7)
Eu colocaria um animal na rua se não tivesse condições para o manter. (1)			
Eu gostaria de colaborar com um abrigo para animais abandonados. (2)			
Eu prefiro comprar animais. (3)			
Os animais adotados são velhos e feios. (4)			
Os animais abandonados sentem-se livres. (5)			
O abandono de um animal é uma prática irresponsável. (6)			
Eu nunca abandonaria o meu animal de companhia. (7)			
Os animais têm de ser protegidos pela lei. (8)			
É irresponsável manter um animal que não se adapte a nós. (9)			
Há animais que são mais felizes com outros animais num abrigo. (10)			
Nenhum animal merece ser separado da sua família humana. (11)			
Há circunstâncias familiares que obrigam a abdicar do animal. (12)			
As associações de proteção animal têm a obrigação de acolher animais indesejados. (13)			
Eu faria de tudo para não ter de abdicar do meu animal. (14)			
O estado tem de se responsabilizar pelos animais indesejados. (15)			
Às vezes não há nada que se possa fazer para manter o animal. (16)			
Só os rafeiros são abandonados, os animais de raça não. (17)			
Não vejo problema algum em abandonar um animal. (18)			
O abandono de um animal nunca tem justificação. (19)			

Start of Block: confiança

Q88 Para cada uma das 6 afirmações que se seguem, assinale como se posiciona:

	Discordo totalmente 1 (1)	2 3 4 5 6	Concordo totalmente 7 (7)
Normalmente, os animais de companhia são sinceros. (1)			
Normalmente, os animais de companhia são dignos de confiança. (2)			
Normalmente, os animais de companhia são bondosos e amáveis. (3)			
Normalmente, as outras pessoas confiam nos animais de companhia. (4)			
Os animais de companhia podem confiar em mim. (5)			
Normalmente, os animais de companhia são amáveis quando os outros confiam neles (6)			

Supplementary Material

	PORTUGUESE QUESTIONNAIRE	MEANING IN ENGLISH	ORIGINAL
Q72	Quais os motivos que o/a levariam a abdicar do seu animal?	What motives would lead you to relinquish your animal?	Based on the main reasons for relinquishment reported by Salman et al. [1]
	Não ter solução para as férias (1)	Not having a solution for the holidays	(created by the authors)
	Doença dispendiosa do animal (2)	Expensive animal illness	Ill<br>Euthanaqia-illness
	Idade avançada (3)	Old age	Too old<br>Euthanasia-old age
	Comportamentos agressivos (4)	Aggressive behaviors	Aggressive to people<br>Aggressive to animals
	Comportamentos destrutivos (5)	Destructive behaviors	Outside destruction<br>Inside destruction
	Urina ou fezes fora do sítio (6)	Urine or feces misplaced	House soiling
	Eu ou familiar alérgico ao animal (7)	Me or a family member allergic to the pet	Allergies
	Personalidade do animal incompatível comigo (8)	Animal’s personality incompatible with me	Afraid<br>Not friendly<br>Too mellow
	Personalidade do animal incompatível com a família (9)	Animal’s personality incompatible with my family	Children incompatible<br>New baby<br>Incompatible with other pets
	Comportamento do animal incompatível comigo (10)	Animal’s behavior incompatible with me	Too vocal<br>Too active<br>Jumps on people
	Comportamento do animal incompatível com a família (11)	Animal’s behavior incompatible with my family	Children incompatible<br>New baby<br>Incompatible with other pets
Q75	Já alguma vez teve de abdicar de um animal porque não o podia manter? O que aconteceu?	Have you ever relinquished a pet because you couldn’t keep them? What happened?	(created by the authors)
	Nunca aconteceu (1)	Never happened	
	Sim, entreguei o animal a um familiar/amigo (2)	Yes, I surrendered the animal to a family member/friend	
	Sim, devolvi o animal à origem (criador/associação/particular) (3)	Yes, I returned the animal to their origin (breeder/shelter/family)	
	Sim, vendi o animal (4)	Yes, I sold the animal	
	Sim, soltei o animal no exterior (5)	Yes, I released the animal outdoors	
	Sim, o animal foi eutanasiado (6)	Yes, the animal was euthanized	
	Sim, entreguei a uma associação de proteção animal (7)	Yes, I surrendered the animal to a shelter	
	Outro: (8)	Other	

Q85	Leia estas afirmações e indique o quanto concorda ou discorda de cada uma:	Read these statements and choose how much you agree or disagree with each of them	Adapted from Mazas et al. [2]
	Eu colocaria um animal na rua se não tivesse condições para o manter. (1)	I would place an animal on the street if I didn't have the conditions to keep it.	I would leave an animal in the countryside if I got bored of it
	Eu gostaria de colaborar com um abrigo para animais abandonados. (2)	(used verbatim)	I would love to collaborate with a shelter for abandoned animals
	Eu prefiro comprar animais. (3)	I prefer to buy animals.	I always buy pets from pet shops; the ones in animal shelters are old and ugly
	Os animais adotados são velhos e feios. (4)	Adopted animals are old and ugly.	
	Os animais abandonados sentem-se livres. (5)	(used verbatim)	Abandoned animals feel free
	O abandono de um animal é uma prática irresponsável. (6)	Abandoning a animal is an irresponsible practice	Animal abandoning is a very cowardly and irresponsible practice
	Eu nunca abandonaria o meu animal de companhia. (7)	(used verbatim)	I would never abandon my pet
	Os animais têm de ser protegidos pela lei. (8)	(used verbatim)	Animals must be protected by law
	É irresponsável manter um animal que não se adapte a nós. (9)	It's irresponsible to keep a pet that doesn't adjust to us.	(created by the authors)
	Há animais que são mais felizes com outros animais num abrigo. (10)	There are animals that are happier with other animals in a shelter.	
	Nenhum animal merece ser separado da sua família humana. (11)	No animal deserves to be separated from their human family.	
	Há circunstâncias familiares que obrigam a abdicar do animal. (12)	There are family circumstances that force the relinquishment of the animal.	
	As associações de proteção animal têm a obrigação de acolher animais indesejados. (13)	Animal protection organizations are obliged to take unwanted animals.	
	Eu faria de tudo para não ter de abdicar do meu animal. (14)	I would do anything to not have to relinquish my animal.	
	O estado tem de se responsabilizar pelos animais indesejados. (15)	The state must be responsible for unwanted animals.	
	Às vezes não há nada que se possa fazer para manter o animal. (16)	Sometimes there's nothing that can be done to keep the animal.	
	Só os rafeiros são abandonados, os animais de raça não. (17)	Only mutts are abandoned, breed animals are not.	
	Não vejo problema algum em abandonar um animal. (18)	I don't see any problem in abandoning an animal.	
	O abandono de um animal nunca tem justificação. (19)	The abandonment of an animal is never justified.	
Q88	Para cada uma das 6 afirmações que se seguem, assinale como se posiciona:	For each of the following statements, choose what's your position:	Adapted from Yamagishi and Yamagishi [3]
	Normalmente, os animais de companhia são sinceros. (1)	Usually, pets are sincere.	Most people are basically honest.

Normalmente, os animais de companhia são dignos de confiança. (2)	Usually pets are trustworthy.	Most people are trustworthy.
Normalmente, os animais de companhia são bondosos e amáveis. (3)	Usually pets are kind and lovely.	Most people are basically good and kind.
Normalmente, as outras pessoas confiam nos animais de companhia. (4)	Usually, most other people trust in pets.	Most people are trustful of others.
Os animais de companhia podem confiar em mim. (5)	Pets can trust me.	I am trustful.
Normalmente, os animais de companhia são amáveis quando os outros confiam neles (6)	Normally pets are kind when others trust in them.	Most people will respond in kind when they are trusted by others.

References:

1. Salman, M.D.; New Jr., J.C.; Scarlett, J.M.; Kass, P.H.; Ruch-Gallie, R.; Hetts, S. Human and Animal Factors Related to Relinquishment of Dogs and Cats in 12 Selected Animal Shelters in the United States. *J. Appl. Anim. Welf. Sci.* **1998**, *1*, 207–226 https://doi.org/10.1207/s15327604jaws0103_2.
2. Mazas, B.; Manzanal, M.R.F.; Zarza, F.J.; María, G.A. Development and Validation of a Scale to Assess Students' Attitude towards Animal Welfare. *Int. J. Sci. Educ.* **2013**, *35*, 1775–1799 https://doi.org/10.1080/09500693.2013.810354.
3. Yamagishi, T.; Yamagishi, M. Trust and Commitment in the United States and Japan. *Motiv. Emot.* **1994**, *18*, 129–166 https://doi.org/10.1007/BF02249397.
